# Crocetin Protected Human Hepatocyte LO2 Cell From TGF-β-Induced Oxygen Stress and Apoptosis but Promoted Proliferation and Autophagy *via* AMPK/m-TOR Pathway

**DOI:** 10.3389/fpubh.2022.909125

**Published:** 2022-06-28

**Authors:** Hongxing Guo, Chenyu Ruan, Xiuhong Zhan, Hao Pan, Yumei Luo, Ke Gao

**Affiliations:** ^1^Department of Gastroenterology, The Fifth Affiliated Hospital, Southern Medical University, Guangzhou, China; ^2^Department of Pathology, Foshan Fosun Chancheng Hospital, Foshan, China

**Keywords:** crocetin, proliferation, autophagy, apoptosis, inflammation, AMPK/m-TOR

## Abstract

**Objective:**

To investigate the protective effects of crocetin against transforming growth factor-β (TGF-β)—induced injury in LO2 cells.

**Methods:**

Human hepatocyte LO2 cells were pre-treated with crocetin (10 μM) for 6, 12, and 24 h, and then induced by TGF-β. Proliferation, oxidative stress, apoptosis, autophagy, and related proteins were assessed.

**Results:**

Crocetin pre-treating promoted proliferation but suppressed apoptosis in TGF-β-induced LO2 cells. Crocetin protected LO2 cells from TGF-β-induced inflammation and oxygen stress by reducing reactive oxygen species (ROS) and malondialdehyde (MDA) but enhancing superoxide dismutase (SOD) and glutathione (GSH). Autophagy was suppressed in TGF-β but crocetin promoted autophagy in LO2 cells by mediating Adenosine 5'-monophosphate—activated protein kinase (AMPK)/mammalian target of rapamycin (m-TOR) signaling pathway *via* upregulating p-AMPK and p-Beclin-1 but downregulating p-mTOR.

**Conclusions:**

Crocetin protected LO2 cells from TGF-β-induced damage by promoting proliferation and autophagy, and suppressing apoptosis and anti-inflammation *via* regulation of AMPK/m-TOR signaling pathway.

## Introduction

Saffron is a perennial herb of *Crocus sativus* (*Iridaceae*), which is not only a kind of spice but also a highly valued medicine (1, 2). As a component of traditional medicine, saffron had been used as a medicinal herb for treating various ailments including cramps, asthma, liver disease, menstruation disorders, pain, and in the pathogenesis of cancer (3–8). Crocin, crocetin, picrocrocin and safranal are the main secondary metabolites of Crocus sativus (9). These metabolites are responsible for the color, bitter taste, odor, and aroma (9). A number of pharmacological studies have demonstrated that crocetin (8,8′-diapocarotene-8,8′-dioic acid) has a wide range of activities, including antioxidant, anti-cancer, hypolipidemic, and anti-atherosclerotic effects.

The liver is a major site of xenobiotic metabolism. It can be injured by toxic chemicals, drugs, and virus infiltration from ingestion or infection (10). In the case of the liver, TGF-β signaling participates in all stages of disease progression, from initial liver injury through inflammation and fibrosis, to cirrhosis and cancer (11–13). TGF-β has cytostatic and apoptosis effects on hepatocytes, as well as promoting liver differentiation during embryogenesis and physiological liver regeneration (14). However, a high level of TGF-β is the consequence of chronic liver damage, resulting in the activation of stellate cells into myofibroblasts and massive hepatocyte cell death. This process contributes to the promotion of liver fibrosis and later cirrhosis. Notably, cumulative evidence illustrates that the anti-inflammatory and immunomodulatory properties of crocetin have been comprehensively examined in various diseases characterizing liver injury and apoptosis (15, 16). However, the mechanisms of crocetin for reducing apoptosis have not yet been evaluated clearly.

In our previous research, we explored the effects of crocetin against lipopolysaccharide (LPS)/D-galactosamine (DGalN) induced—fulminant hepatic failure (FHF) rats (17). The results showed the protective effects on FHF, as well as the suppression of apoptosis, inflammation, and oxidative stress, which were related to the apoptosis proteins in the caspase family and Bcl-2 family, and the modulation of the nuclear factor κ-B (NF-κB) signal pathway. microRNAs also play important roles as they participate in cellular regulation. We found that miR-224 might hinder the hepatitis B virus (HBV) replication by attenuating SIRT1-mediated autophagy, indicating the correlation between liver diseases and autophagy in hepatocytes (18).

In this study, we indicated the therapeutic potentials of crocetin on liver injury using a LO2 cell line, which was pre-treated with crocetin at different times, and then induced by TGF-β for establishing a liver injury model. Related expressions of inflammation and stress reaction were investigated. Apoptosis and autophagy were studied by measuring the activity of the AMPK/m-TOR signaling pathway. This study provides insights into the potential medical treatment of liver injury with theoretical mechanisms.

## Materials and Methods

### Drug and Reagents

Crocetin was obtained from MP Biomedicals, CA, United States. Crocetin was purified by high-performance liquid chromatography (HPLC) with 98% purity and then dissolved in dimethyl sulfoxide (DMSO; Sigma-Aldrich, Merck KGaA, Darmstadt, Germany) with a 0.1% final concentration in the culture medium. TGF-β was purchased from Merck Millipore (Burlington, MA, United States). Antibodies including rabbit anti-Caspase3 (#2723), mouse anti-Bcl-2 (#15071), mouse anti-Bax (#89477), rabbit anti-TGF-β (#3711), rabbit anti-α-SMA (#19245), rabbit anti-p-AMPKα (#50081), rabbit anti-p-Beclin-1 (#35955), mouse anti-SIRT-1 (#8469), rabbit anti-LC3 (#12741), and rabbit anti-NDRG2 (#5667) were purchased from Cell Signaling Technology (CST) Inc., Beverly, MA, United States. Mouse anti-GAPDH (sc-365062) and mouse anti-p-mTOR (sc-293113) were obtained from Santa Cruz Biotechnology, Inc., CA, United States.

### Cell Culture

LO2 cells were purchased from the American Type Culture Collection (ATCC, Manassas, VA, United States). Cells were cultured with DMEM (Thermo Fisher, Scientific, Inc., Waltham, MA, United States) containing 10% fetal bovine serum (FBS; Gibco, Thermo, Sydney, NSW, Australia), and maintained at 37°C, 5% CO_2_, and saturated humidity. Before treatment, cells were starved for 24 h. Before TGF-β stimulation, cells were pre-treated with 10 μM of crocetin for 6, 12, and 24 h, respectively. After pret-reating, LO2 cells were incubated with 2 ng/ml of TGF-β (Merck Millipore) in DMEM supplemented with 1% FBS for 24 h. The cells in stable growth were used in the following experiments.

### Cell Viability Assay

Cells were cultured in the 96-well plate (5,000 cells/well), six repetitions of each. The LO2 cells were treated as mentioned before. 10 μl of the Cell Counting Kit-8 (CCK-8; Thermo) solution was added to each well and then incubated at 37°C for 4 h. The optical density (OD) value at 450 nm was measured by a multi-plate reader (BioTek, Winooski, VT, United States). Experiments were repeated three times.

### Flow Cytometry

Apoptosis was analyzed by double-staining with Annexin V-FITC and PI (BD Biosciences Inc., San Jose, CA, United States). The LO2 cells were treated as mentioned before. The cells were collected and washed twice with pre-cooling phosphate-buffered saline (PBS, Thermo), and then adjusted to a concentration of 1 × 10^6^ cells/ml. Resuspended cells in 500 μl of PBS buffer containing 5 μl of Annexin V-FITC and 10 μl of PI, and then incubated at room temperature in the dark for 15 min. The apoptosis cells were analyzed by a FACS Calibur Flow Cytometer (Becton, Dickinson and Company, CA, United States) within 1 h.

### Biochemical Analysis

The LO2 cells were treated as mentioned before. Each group of cells was collected for measuring the activities of ROS, MDA, SOD, and GSH. ROS, MDA, SOD, and GSH were measured according to the specification of the assay kits (Nanjing Jiancheng Institute of Biotechnology, Nanjing, China). The fluorescence of ROS was measured at 525 (530 ± 20 nm). The OD values of MDA, SOD, and GSH were detected at 532, 420, and 405 nm, respectively.

### Western Blotting Analysis

The LO2 cells were treated as mentioned before. Cells were collected after 48 h and washed twice with pre-cooling PBS. The cells were lysed with radio-immunoprecipitation (RIPA) buffer (Beyotime Biotechnology, Shanghai, China) containing 1% phenylmethylsulfonyl fluoride (PMSF, Beyotime). After incubation for 30 min on ice, the cell lysates were centrifuged at 12,000 × g for 10 min, and the supernatants were collected. BCA protein assay (Pierce, Rockford, IL, United States) was used to quantify the protein content. Proteins were separated by sodium dodecyl sulfonate - polyacrylamide (SDS-PAGE) with 10% separated gel and 5% stacking gel, and then transferred into polyvinylidene fluoridemembranes (PVDF, Merck Millipore) membrane. After blocking with 5% skimmed milk, the membrane was cut into protein bands and then incubated with primary antibodies at 4°C, overnight. After being washed with 0.1% Tween-20 in PBS (PBST), bands were incubated with IRDye800® Conjugated secondary antibody (1:3,000, Rockland, American) at room temperature for 1 h. With electrochemiluminescence (ECL, Thermo) assay, bands were scanned and analyzed with Odyssey Infrared Imaging System (LI-COR Biosciences, Manassas, VA, United States). The integrated intensity for each band was determined with Odyssey Imager Software (v3.0). The experiments were repeated at least three times.

### Transmission Electron Microscope Analysis on Autophagy

The LO2 cells were treated as mentioned before. Cells were fixed in 2.5% of glutaraldehyde (Solarbio Life Sciences Co., Ltd., Beijing, China) in PBS for 2 h, and then fixed in 2% of osmic acid (Merck Millipore) in PBS for 2 h. Cells were dehydrated by gradual ethanol: 50% for 15 min, 70% overnight, 80% for 15 min, 90% for 15 min, and 100% for 20 min for three times. Replaced with acetone (Merck Millipore) for 15 min twice. Cells were embedded with acetone in Epon812 (1:2, Merck Millipore) for 4 h, and then embedded with Epon812 for 4 h twice. Following the embedding, polymerized the cells at 65°C for 48 h, and then sectioned into 50~70 mm slices. Stained with 3% uranium acetate and lead nitrate (Merck Millipore) and then observed with TEM (OPTON Talos F200X S/TEM, Beijing, China).

### Statistical Analysis

All data were presented as mean ± S.D., and were analyzed using the SPSS 16.0 program (SPSS, Inc., Chicago, IL, United States). Two-way ANOVA was applied for analyzing data, followed by a *post-hoc* test with Fisher's least significant difference (LSD) to determine the differences among groups. A value of *P* < 0.05 was considered statistically significant.

## Results

### Crocetin Affect Cell Viability and Cell Apoptosis

**Figure 1:**
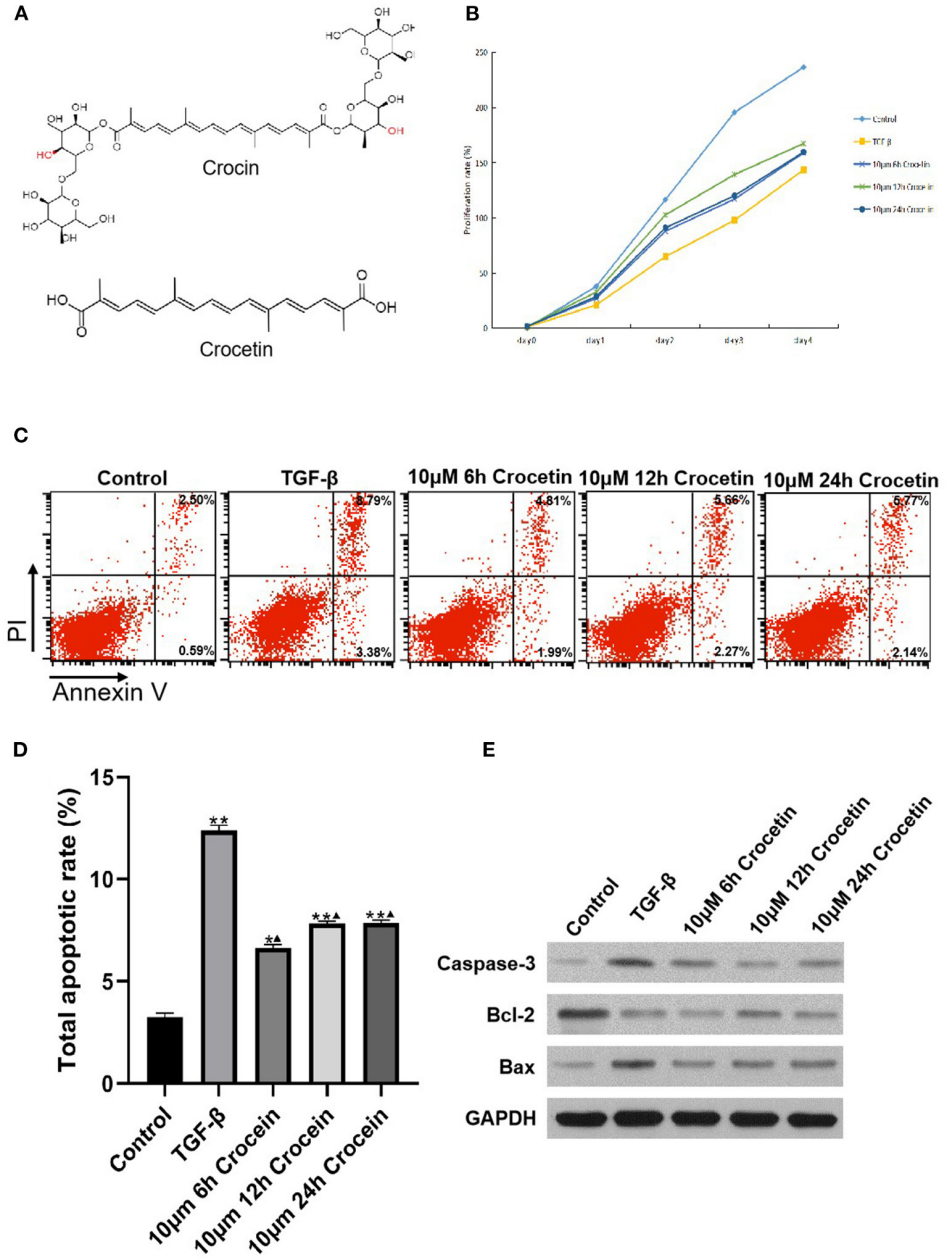
LO2 cells were incubated with 10 μM of crocetin for 6, 12, and 24 h, respectively, and then induced by 2 ng/ml of TGF-β. Cell viability was measured by CCK-8 and apoptosis was by flow cytometry. **(A)** Chemical structure of crocetin. **(B)** Cell viability was detected by CCK-8 assay. **(C)** Cell apoptosis was measured by flow cytometry. **(D)** Cell apoptosis was compared among the groups with or without crocetin pre-treatment and/or TGF-β induction. **(E)** Apoptosis proteins Caspase-3, Bcl-2, and Bax levels in LO2 cells were measured by Western Blotting. *Comparing to the Control, *P* < 0.05. **Comparing to the Control, *P* < 0.01. ^▴^Compared to TGF-β, *P* < 0.05.

Using the CCK-8 assay, we found that cell viability was suppressed with the stimulation of TGF-β in LO2 cells (Figures 1A,B). In comparison, the cell viability was improved in the crocetin administrative group, which means crocetin might protect LO2 cells from TGF-β-induced injury. To verify this, cell apoptosis was evaluated using a double-staining with Annexin V-FITC/PI by flow cytometry. The results showed that LO2 cells with TGF-β stimulation had a higher apoptosis rate than the normal cells (Figures 1C,D). Moreover, crocetin significantly inhibited the TGF-β-induced apoptosis in LO2 cells in a time-dependent manner. The 6 h group had better efficiency in inhibiting LO2 cells apoptosis than that of 12 and 24 h groups. In addition, apoptosis-related proteins, Caspase-3, Bcl-2, and Bax were tested. TGF-β increased the levels of caspase-3 and Bax but decreased the level of Bcl-2 in LO2 cells in comparison with the control group (Figure 1E). In contrast, as the time during which the crocetin was pre-treated increased, the caspase-3 level decreased gradually and obviously. Bax level decreased in both the crocetin treating groups but without obvious time-dependence. The Bcl-2 level increased during crocetin pre-treating for the 12 h group, but this was not obvious for crocetin pre-treating in the 6 and 24 h groups. Our findings suggested that crocetin could attenuate TGF-β-induced cell apoptosis and the viability in LO2 cells.

### Crocetin Suppress Oxidative Stress and Inflammation

**Figure 2:**
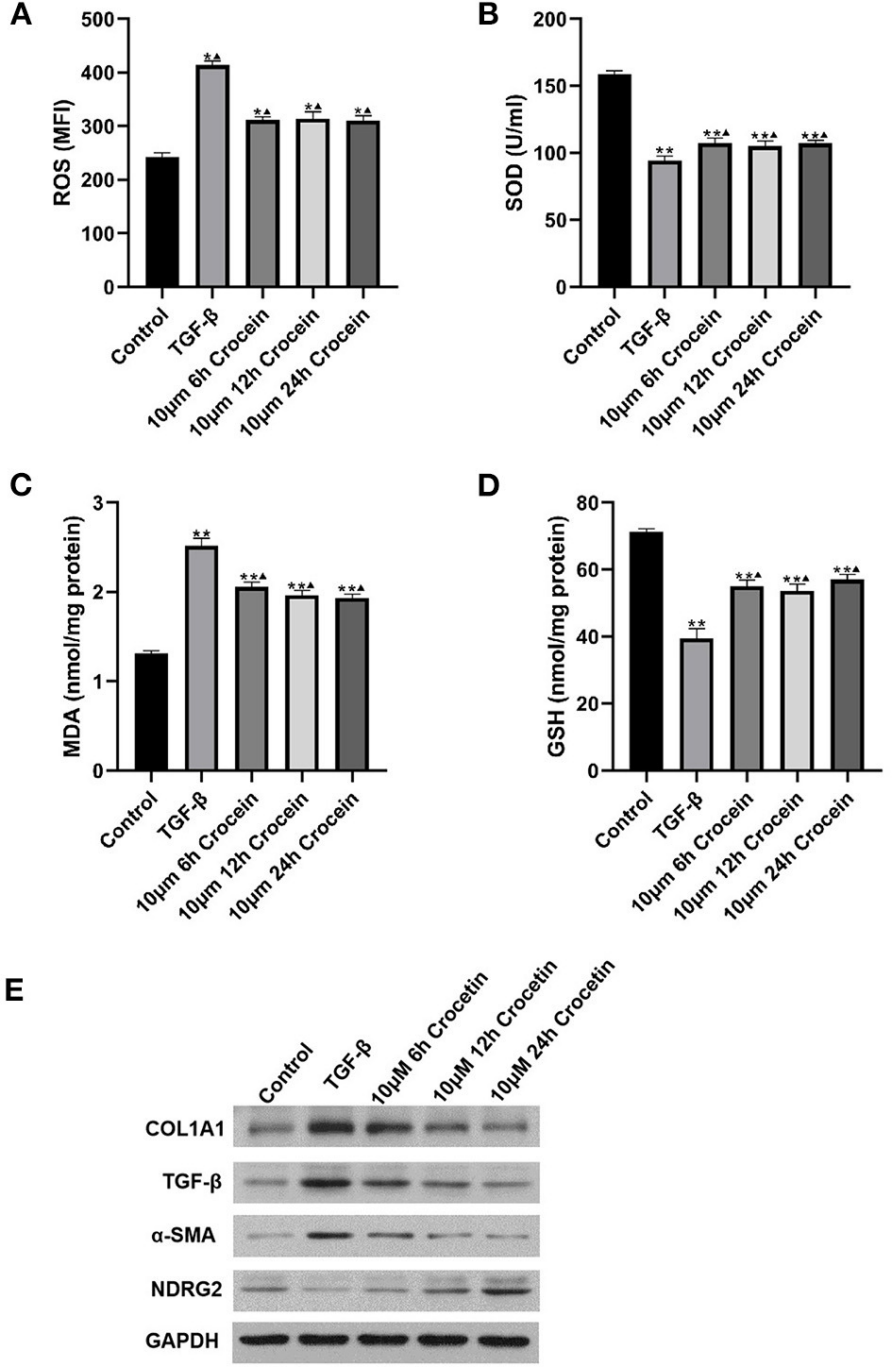
LO2 cells were incubated with 10 μM of crocetin for 6, 12, and 24 h, respectively, and then induced by 2 ng/ml of TGF-β. **(A–D)** Comparisons on ROS, SOD, MDA, and GSH levels among the groups with or without crocetin pre-treatment and/or TGF-β induction. **(E)** Expressions of COL1A1, TGF-β, α-SMA, and NDRG2 in LO2 cells were measured by western blot. *Comparing to the Control, *P* < 0.05. ** Comparing to the Control, *P* < 0.01. ^▴^Compared to TGF-β, *P* < 0.05.

To assess the anti-oxidative effects of crocetin, the levels of ROS, SOD, MDA, and GSH were measured, and the results are shown in Figure 2. Figure 2A shows that the ROS level was elevated in the TGF-β-induced group compared to the control. With crocetin pre-treatment for different times, ROS decreased significantly, compared to the TGF-β group, but was still higher than in the control. As shown in Figure 2B, treatment with TGF-β resulted in a significant decrease in SOD level compared to the control. Pre-treating with crocetin increased the level of SOD compared to the TGF-β group but was still lower than the control. Figure 2C showed that the MDA level was elevated in the TGF-β-induced group compared to the control. Crocetin pre-treatment for different times decreased MDA level significantly, compared to the TGF-β group, but was still higher than the control. Figure 2D showed that the GSH level was reduced in the TGF-β-induced group compared to the control. Crocetin pre-treatment enhanced GSH level compared to the TGF-β group but was still lower than the control.

In general, TGF-β treatment increased reactive oxygen in response to inflammatory agonists. To explore the mechanism, COL1A1, TGF-β, α-SMA, and NDRG2 were analyzed by Western Blotting. Experimental results suggested that COL1A1 and α-SMA protein levels were markedly higher after TGF-β-induction compared to the control (Figure 2E). COL1A1 and α-SMA also decreased gradually and significantly, showing in a time-dependent manner with crocetin pre-treatment. In contrast, NDRG2 was reduced after TGF-β-induction compared to the control, but enhanced with crocetin pre-treatment in a time-dependent manner. These results indicated that crocetin prevented LO2 cells from TGF-β-induced injury by regulating ROS, SOD, MDA, and GSH levels, as well as COL1A1, TGF-β, α-SMA, and NDRG2 levels.

### Crocetin Promotes Cell Autophagy *via* AMPK/m-TOR Signaling Pathway

**Figure 3:**
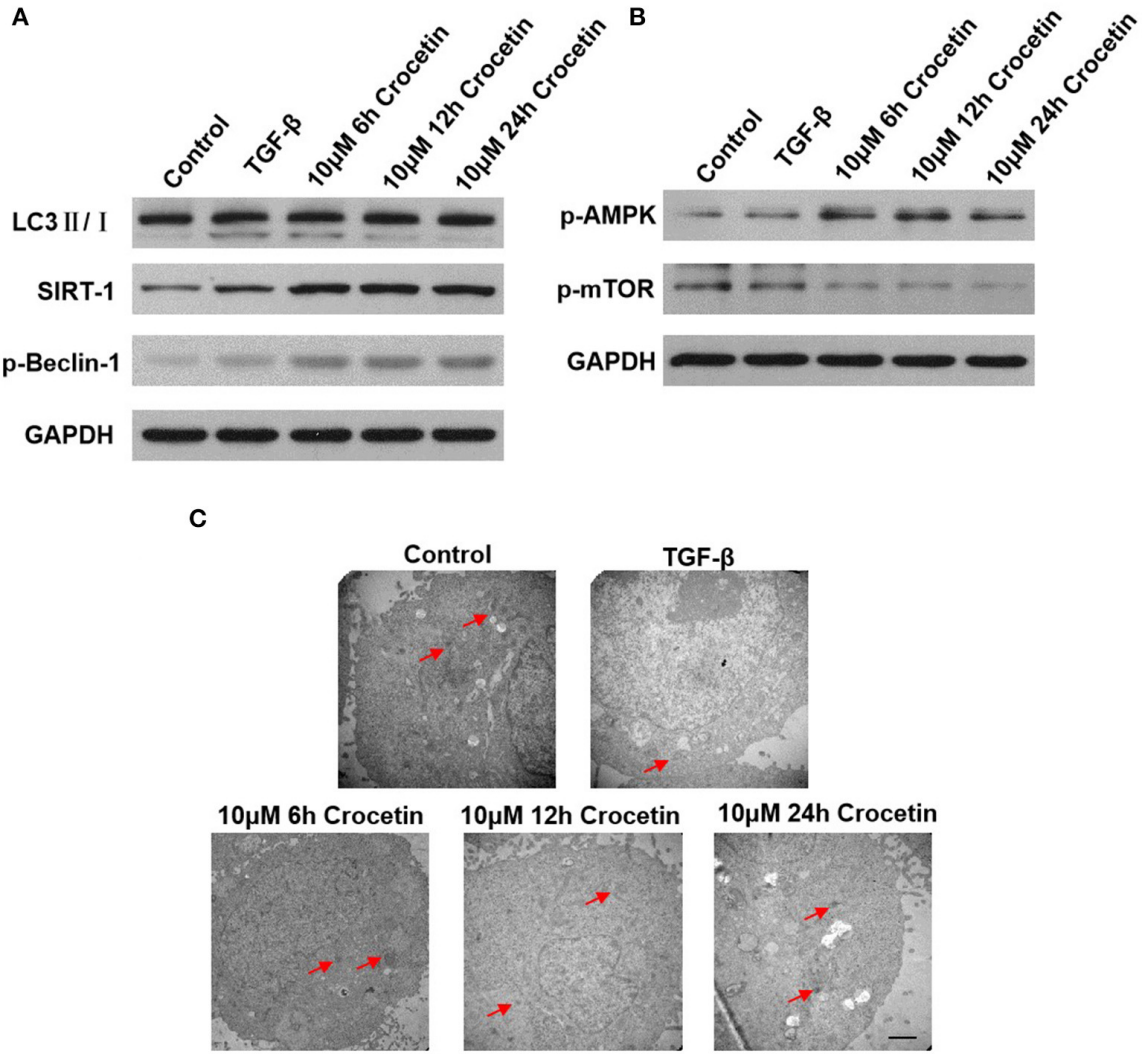
Crocetin pre-treatment promoted LO2 cell autophagy *via* regulating the AMPK/m-TOR signaling pathway. **(A)** The level of SIRT-1, LC3II/I, and Beclin-1 were examined by western blot. **(B)** AMPK/m-TOR signaling pathway-related protein was assessed by Western Blot. **(C)** The autophagy process was observed using TEM analysis.

To investigate the effect of crocetin on autophagy in TGF-β-induced injury in LO2 cells, we first measured the level of LC3II/I. As Figure 3A shows, the LC3II/I ratio was elevated in LO2 cells after being pre-treated with crocetin, compared to the TGF-β group. The increased LC3II/I presented in a time-dependent manner, significantly. However, the SIRT-1 level was enhanced after TGF-β induction, even presenting at a higher level with crocetin pre-treatment.

By researching the AMPK/m-TOR signaling pathways, the results showed a weakening effect on the m-TOR signal but a promoting effect on AMPK (Figure 3B). Compared to the control, the TGF-β-induction promoted p-AMPK and p-Beclin-1 levels but reduced the p-mTOR level. However, crocetin pre-treatment further improved p-AMPK and p-Beclin-1 levels, but reduced p-mTOR levels.

The autophagy process was observed by TEM analysis (Figure 3C). In the control group, less autophagy was observed. TGF-β-induction enhanced autophagy. With crocetin pre-treatment, autophagy was promoted, showing that the number of autophagosomes and autophagy lysosomes were significantly greater than in the control.

## Discussion

This study used human hepatocyte LO2 cells as the model cell. Crocetin was the protected agent used to pre-treat LO2 cells before TGF-β-induced injury. Cell proliferation, apoptosis, oxygen stress, inflammation, and autophagy were measured to analyze the protective effect of crocetin. The results showed that different pre-treating times of crocetin (10 μM) could prevent LO2 cells from TGF-β-induced injury, presenting as promoting cell proliferation and autophagy, and suppressing apoptosis and oxygen stress. This protective effect might occur by regulation of the AMPK/m-TOR signaling pathway.

In recent years research interests have increasingly focused on complementary and alternative medicines for treating various diseases. Crocetin is a member of carotenoid compounds with antioxidants. Tseng et al. (19) have suggested that crocetin could attenuate ROS-induced hepatotoxicity and genotoxicity *via* quenching of the superoxide anion and/or free-radicals. Zheng et al. (20) used hypercholesterolemic rabbits as the model and found that crocetin contributed to the attenuation of atherosclerosis by suppressing the expression of vascular cell adhesion molecule-1 (VCAM-1). Wang et al. (21) suggested that crocetin showed a protective effect on acute renal failure rats induced by hemorrhagic shock. A large number of other studies have confirmed the protective effect of crocetin on the liver. Chen et al. (22) confirmed the protective effect of crocetin on CCL4-induced liver injury in mice. Dhar et al. (23) used hemorrhagic shock mice to confirm the protective effect of crocetin on survival and liver tissue damage. Sreekanth et al. (24) found that crocetin could improve dengue virus-induced liver injury but could not suppress virus replication. As a traditional drug, crocetin is considered to have benefits on the nervous system, protective effects from cardiovascular disease, antioxidation, and so on.

Our study used TGF-β to induce injury in LO2 cells to imitate the liver injury *in vitro*. Crocetin was pre-treated before TGF-β-induction, for investigating its protective effect. Firstly, we detected the proliferation of LO2 cells. The results showed the obvious promotion of crocetin on LO2 cell proliferation. Apoptotic LO2 cells were analyzed after TGF-β-induction. The results showed that TGF-β promoted LO2 cell apoptosis and the crocetin pre-treating could suppress LO2 cell apoptosis induced by TGF-β in a time-dependent manner. Inhibition of 6 h of pre-treating time had a better effect on apoptosis than 12 and 24 h. According to previous literature, crocetin has been found to inhibit the proliferation and promote the apoptosis of tumor cells such as pancreatic cancer MIA-PaCa-2 cell (25), MG63 myeloma cells (26), colon cancer cells (27), gastric cancer cells (28), and so on. Wang et al. (29) found that crocetin could inhibit the proliferation, migration, and epithelial mesenchymal transition (EMT) induced by TGF-β of retinal pigment epithelial cells. Zhu et al. (30) suggested that crocetin inhibited the proliferation but promoted the apoptosis of hepatic stellate cells (HSC). Our study showed the opposite results to previous research because we used normal liver cell LO2 to establish an inflammatory model. The promotion of proliferation and inhibition of apoptosis confirmed the protective effect of crocetin on normal liver cells.

Secondly, we measured ROS, SOD, MDA, and GSH in LO2 cells with crocetin pre-treatment after TGF-β-induction. The results showed the protective effect of crocetin on maintaining lower levels of ROS and MDA, which were enhanced by TGF-β-induction, as well as the higher levels of SOD and GSH, which were reduced by TGF-β-induction. Niska et al. (31) confirmed the protective effect of crocetin on antioxidant defense and detoxification systems induced by CuO nanoparticles in mouse hippocampal HT22 cells. Shen et al. (32) also found that crocetin might ameliorate norepinephrine-induced injury in cardiac myocytes of the rat by enhancing SOD and GSH but decreasing lipid peroxidation and Ca^2+^. Our results agreed with previous research.

Hepatic fibrosis is the chronic inflammation of the liver. It can lead to the imbalance of collagen proliferation and degradation, finally resulting in cirrhosis (33). COL1A1 and α-SMA are expressed at low levels in a healthy liver, while NDRG2 is expressed at high levels in normal tissues. When with chronic disease or cancer, COL1A1 and α-SMA were enhanced, but NDRG2 was suppressed. Thirdly, we used TGF-β to induce LO2 injury, leading to the increasing COL1A1 and α-SMA but decreasing NDRG2 levels. Crocetin pre-treating suppressed the increasing COL1A1 and α-SMA and decreased NDRG2 induced by TGF-β. These results suggested that crocetin pre-treatment prevented hepatic fibrosis and cellular inflammation.

Autophagy refers to the formation of autophagosomes. When some organelles and proteins need to be degraded, the bilayer membrane from the rough endoplasmic reticulum (ER) without ribosome wraps them and then combines with the lysosome to form autolysosome. Autolysosome is helpful for the cellular metabolism and renewal of organelles (34). In our studies, we detected the autolysosome in LO2 cells with crocetin pre-treatment and TGF-β induction. The results showed the obvious inhibition of autolysosomes' formation in TGF-β-induced LO2 cells. However, crocetin pre-treatment promoted autophagy in TGF-β-induced LO2 cells and increased autolysosome formation. Decreasing SIRT-1, induced by TGF-β, was also enhanced by crocetin pre-treating. SIRT-1 was the metabolic sensor and the transcriptional regulator, which suppressed inflammatory related factors inducible NO synthase and intercellular adhesion factor 1 (ICAM1) (34). LC3 was deacetylated by SIRT-1, resulting in the enhancing LC3II and decreasing LC3I. Beclin-1 combined with autophagy pre-cursor and promoted the formation of autolysosome. All of these proteins represented the promotion of autophagy by crocetin pre-treatment.

To investigate how crocetin protects LO2 cells from TGF-β, we measured the AMPK/m-TOR signal pathway. The results showed that p-AMPK was significantly enhanced and decreased p-mTOR in crocetin pre-treating LO2 cells. AMPK signal pathway is the critical regulator of bioenergy metabolism, while m-TOR plays an essential role in the growing, aging, and cancer genesis of many diseases of organisms. Overexpression of m-TOR represents disease generation (35, 36). Inducing TGF-β resulted in the injury of LO2 cells, suppressing AMPK but promoting m-TOR signals. Crocetin might protect LO2 cells from TGF-β-induced injury by promoting AMPK and suppressing overexpression of m-TOR to balance cellular metabolism and growth.

## Conclusions

In this study, we used TGF-β to induce injury in LO2 cells as a liver damage model *in vitro*. Crocetin was used as the protective agent to pre-treat LO2 cells before TGF-β induction. With crocetin pre-treatment, LO2 cell proliferation was promoted and apoptosis was inhibited. Antioxidation was enhanced and hepatic fibrosis was suppressed. Autophagy was promoted by crocetin pre-treatment, which is important for LO2 cell metabolism. The AMPK/m-TOR signal pathway was measured to confirm the cellular mechanisms of crocetin preventing LO2 cells from TGF-β-induced injury. Our results provided clinical prevention and treatment of the occurrence of liver disease with molecular mechanisms.

## Data Availability Statement

The datasets presented in this study can be found in online repositories. The names of the repository/repositories and accession number(s) can be found in the article/supplementary material.

## Author Contributions

KG is the guarantor of the integrity of the entire study and was responsible for the study concepts, study design, and manuscript review. HG was responsible for the definition of the intellectual content of the paper and manuscript preparation and editing. XZ was responsible for literature research and statistical analysis. HP is responsible for clinical data collection. CR was responsible for the experimental studies and data analysis. YL was responsible for data acquisition. All authors contributed to the article and approved the submitted version.

## Funding

This work was supported by the Traditional Chinese Medicine Bureau of Guangdong Province under Grant No. 20201234 and the Natural Science Foundation of Guangdong Province under Grant No. 2018A030310479.

## Conflict of Interest

The authors declare that the research was conducted in the absence of any commercial or financial relationships that could be construed as a potential conflict of interest.

## Publisher's Note

All claims expressed in this article are solely those of the authors and do not necessarily represent those of their affiliated organizations, or those of the publisher, the editors and the reviewers. Any product that may be evaluated in this article, or claim that may be made by its manufacturer, is not guaranteed or endorsed by the publisher.
